# Phylodynamic and Phylogeographic Profiles of Subtype B HIV-1 Epidemics in South Spain

**DOI:** 10.1371/journal.pone.0168099

**Published:** 2016-12-21

**Authors:** Santiago Pérez-Parra, Natalia Chueca, Marta Álvarez, Juan Pasquau, Mohamed Omar, Antonio Collado, David Vinuesa, Ana B. Lozano, Gonzalo Yebra, Federico García

**Affiliations:** 1 Servicio de Microbiología Clínica, Hospital Universitario San Cecilio, Complejo Hospitalario e Instituto de Investigación IBS, Granada, Spain; 2 Servicio de Infecciosas, Hospital Virgen de las Nieves, Granada, Spain; 3 Servicio de Infecciosas, Hospital Ciudad de Jaén, Jaén, Spain; 4 Servicio de Medicina Interna, Hospital de Torrecárdenas, Almería, Spain; 5 Servicio de Infecciosas, Hospital Universitario San Cecilio, Granada, Spain; 6 Servicio de Infecciosas, Hospital de Poniente, Almería, Spain; 7 Institute of Evolutionary Biology, University of Edinburgh, Edinburgh, United Kingdom; Public Health Agency of Canada, CANADA

## Abstract

**Background:**

Since 1982, HIV-1 epidemics have evolved to different scenarios in terms of transmission routes, subtype distribution and characteristics of transmission clusters. We investigated the evolutionary history of HIV-1 subtype B in south Spain.

**Patients & Methods:**

We studied all newly diagnosed HIV-1 subtype B patients in East Andalusia during the 2005–2012 period. For the analysis, we used the reverse transcriptase and protease sequences from baseline resistance, and the Trugene® HIV Genotyping kit (Siemens, Barcelona, Spain). Subtyping was done with REGA v3.0. The maximum likelihood trees constructed with RAxML were used to study HIV-1 clustering. Phylogeographic and phylodynamic profiles were studied by Bayesian inference methods with BEAST v1.7.5 and SPREAD v1.0.6.

**Results:**

Of the 493 patients infected with HIV-1 subtype B, 234 grouped into 55 clusters, most of which were small (44 clusters ≤ 5 patients, 31 with 2 patients, 13 with 3). The rest (133/234) were grouped into 11 clusters with ≥ 5 patients, and most (82%, 109/133) were men who have sex with men (MSM) grouped into 8 clusters. The association with clusters was more frequent in Spanish (p = 0.02) men (p< 0.001), MSM (p<0.001) younger than 35 years (p = 0.001) and with a CD4+ T-cell count above 350 cells/ul (p<0.001). We estimated the date of HIV-1 subtype B regional epidemic diversification around 1970 (95% CI: 1965–1987), with an evolutionary rate of 2.4 (95%CI: 1.7–3.1) x 10^−3^ substitutions/site/year. Most clusters originated in the 1990s in MSMs. We observed exponential subtype B HIV-1 growth in 1980–1990 and 2005–2008. The most significant migration routes for subtype B went from inland cities to seaside locations.

**Conclusions:**

We provide the first data on the phylodynamic and phylogeographic profiles of HIV-1 subtype B in south Spain. Our findings of transmission clustering among MSMs should alert healthcare managers to enhance preventive measures in this risk group in order to prevent future outbreaks.

## 1. Introduction

Human immunodeficiency virus type 1 (HIV-1) is characterised by high genetic variability caused by its high rate of nucleotide substitution [1,2] and recombination capacity [3], which has led to a wide diversity of subtypes and recombinant forms. HIV-1 subtype B is the most prevalent of these variants in central and western Europe [4,5], where it is responsible for almost 70% of new infections [6].

Although the epidemic tends to be stabilising in certain populations [7], in some sub-epidemics, especially in men who have sex with men (MSM), HIV-1 is transmitted more efficiently than in other risk groups [8–11].

Since HIV-1 was identified in the early 1980s [12], molecular epidemiology has helped discover certain aspects of the virus, and has been key in understanding the virus heterogeneity and prevalence of subtypes, and in studying the transmission of drug resistance to antiretroviral drugs [13–17]. Through the phylogenetic relation between viral nucleic acid sequences, phylogenetic analyses can be informative of transmission events between risk groups [18]. Phylodynamics and phylogeography can also help provide data on the evolutionary history of a certain viral population. So they can be used to study the epidemic trends, and the temporal [19–23] and spatial [24–27] dynamics, of this virus.

East Andalusia is located in south Spain, and includes the provinces of Jaén, Almería, and Granada. Tourism and migration in this area have contributed to a wide diversity of HIV-1 viral subtypes, but subtype B is still the most prevalent [28]. Our laboratory is the reference centre for studying antiretroviral drug resistance in this area, and we update a database that links clinical data to virological data. As we have access to the *pol* sequences in all the newly diagnosed patients in this area, we attempted to study the evolutionary history of the HIV-1 subtype B in east Andalusia, and the existence of long-term HIV-1 lineages, by using molecular epidemiology tools. We analysed which groups were at increased risk of infection, the dynamics of viral growth and its spread during the 2005–2012 period.

## 2. Methods

### 2.1 Study population

The analysis included sequences generated from the earliest sample available before ART-initiation (baseline), from all the medical centres that attend HIV-1-infected individuals in east Andalusia, during the 2005–2012 period. The study population included all newly diagnosed HIV-seropositives irrespective of their HIV-infection status (chronic or recent). Of the 693 patients infected with HIV-1 from the provinces of Granada (capital city and Motril), Jaén, and Almería (capital city and El Ejido), 493 (71%) were patients infected with HIV-1 subtype B. Partial *pol* gene sequences (protease (PR), codons 4–99; reverse transcriptase (RT), codons 38–247) were available and linked to demographic (risk group, age, sex, country of origin and attending hospital of origin), clinical (CD4+ T-cell count) and virological (viral load) information.

The Ethics Committee of the San Cecilio Hospital approved the study, and no consent information was required as patient information remained anonymous and was de-identified prior to the analyses. For similar scientific and ethical reasons as explained in other HIV cohorts [10, 29–30], we have submitted a random sample of 10% to GenBank under accession numbers KY110871 to KY110920.

### 2.2 HIV-1 *pol* sequencing and subtype assignment

Protease and reverse transcriptase sequences were obtained with the Trugene® HIV Genotyping kit (Siemens, Barcelona, Spain). The viral subtype was studied with the REGA v3.0 subtyping tool (found at http://dbpartners.stanford.edu:8080/RegaSubtyping/stanford-hiv/typingtool/) and was confirmed by a phylogenetic analysis. A representative data set of the pure subtypes and recombinant forms of HIV-1 group M (A-K + recombinants) sequences from the Los Alamos HIV sequence database (http://www.hiv.lanl.gov) was used as reference.

### 2.3. Phylogenetic Analysis

All the sequences were edited manually and aligned using ClustalW [31]. The best-fit model of nucleotide substitution for the analysis was obtained through FindModel (accessible at http://www.hiv.lanl.gov/content/sequence/findmodel/findmodel.html). A preliminary analysis was performed using maximum likelihood (ML) (*randomised Accelerated Maximum Likelihood*, RAxML), accessible through the CIPRES science Gateway [32]. We used the best-fit model selected by FindModel: general time-reversible (GTR) with gamma-distributed rate heterogeneity across sites model and 1,000 bootstrap iterations for this analysis. The associations between the sequences with related nodes were studied, where a 70% bootstrap value was taken as a significantly reliable value [33]. However, we confirmed these lineages by the Bayesian approach described below. We analysed the subtype B lineages of greater epidemiological relevance in detail (five or more related sequences).

### 2.4 Statistical analyses and clustering predictors

A logistic regression analysis was run to assess the associations among the clustering and clinical, demographic or virological characteristics. Statistical analyses were carried out with SPSS 15 (SPSS Inc., Chicago, IL, USA).

### 2.5 Reconstruction of evolutionary and demographic history

The Bayesian Markov Chain Monte Carlo (MCMC) approach was applied to this data set, as implemented in BEAST v1.7.5 [34,35]. To improve the convergence of chains and molecular clock precision, the BEAST analysis was applied to a large set of the east Andalusian HIV-1 sequences (n = 419). A time scale in phylogenetic trees was set using the Shapiro-Rambaut-Drummond-2006 (SRD06) nucleotide substitution model, which adapts the *Hasegawa*, *Kishino*, *and Yano (HKY*) nucleotide substitution model by applying two partitions according to the nucleotide codon (first/second and third). We estimated the evolutionary rate (μ, nucleotide substitutions per site per year, subst./site/year) by a previous analysis with 200 HIV-1 subtype B sequences from the viruses collected during 27 years, which were retrieved from the Los Alamos HIV sequence database. These data were used to adjust a lognormal prior distribution for the clock rate (ucld.mean parameter; mean = 0.0024, stdev = 0.25).

We used a relaxed uncorrelated lognormal clock (UCLN) [36] and a demographic nonparametric model, Bayesian Skyline Plot (BSP) [37]. The MCMC was run for 250 million states by sampling every 50,000. We estimated the evolutionary rate (μ, nucleotide substitutions per site per year, subst./site/year) and the time and location of the most recent common ancestor (MRCA) of the different HIV-1 clades. For this analysis, we employed TRACER v1.6 (accessible at http://tree.bio.ed.ac.uk/software/tracer/). Only traces with an effective sample size (ESS) of > 200 were accepted after excluding an initial 10%. The BSP model allowed us to estimate effective changes in population size (Ne), and to run a demographic analysis in TRACER v1.6. To estimate the ancestral localisation of the virus and the most significant epidemiological links, we followed a discrete asymmetric Bayesian phylogeographic approach by means of Bayesian Stochastic Search Variable Selection (BSSVS), and by georeferencing each sequence with discrete latitude and longitude data. We used SPREAD V1.0.6 (available at http://www.kuleuven.ac.be/aidslab/phylogeography/SPREAD.html.) and Bayes factor sets to identify well-supported rates and to summarise the geographic spread and the most significant epidemiological links of HIV-1 subtype B dispersal in east Andalusia. All the trees were visualised and edited with FigTree, v 1.4.0 (available at http://tree.bio.ed.ac.uk/software/figtree). In these trees, the clusters of more epidemiologic relevance, as previously defined by ML, and with a posterior probability above 0.9 (PP>0.9), were studied.

## 3. Results

**Fig 1:**
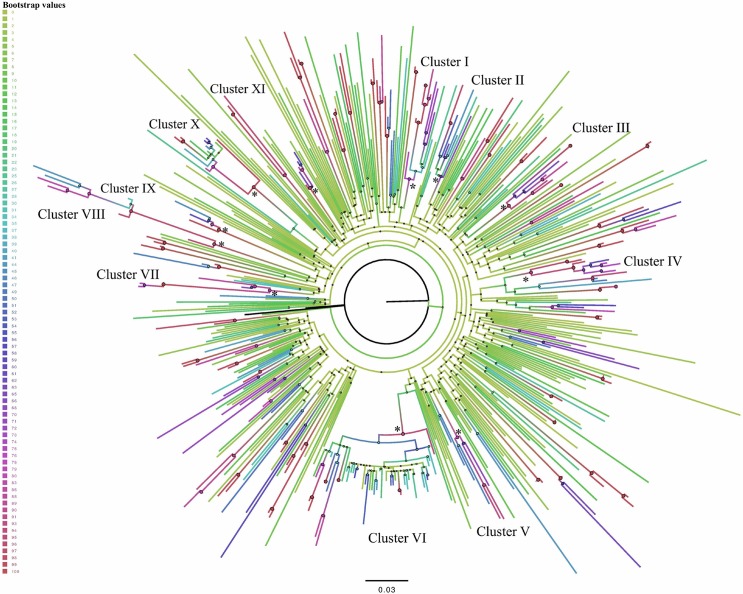
ML phylogenetic tree of the global HIV-1 subtype B *pol* sequences. The phylogenetic tree was constructed by the general time-reversible with gamma-distributed rate heterogeneity across sites model of substitution implemented into RAxML. Branches are drawn on scale with the bar at the bottom, which represents 0.03 nucleotide substitution per site and a progressive bootstrap gradient value using FigTree, v. 1.4. The statistically highly supported nodes (bootstrap >70%) and the clusters with five or more related sequences are indicated by an asterisk (*).

The phylogenetic analyses through ML (Fig 1) showed 55 clusters, which represented 47% (n = 234) of our population: 43% (101/234) of the patients grouped into 44 small clusters: 31 clusters of two patients [13 MSM, 12 heterosexual (HTX), and six intravenous drug users (IVDU)]; 13 clusters of three patients, (MSM = 7, HTX = 5 and IVDU = 1). All the other patients (57% (133/234)) were grouped into 11 clusters of five subjects or more. Their demographic, clinical and virological characteristics are offered in Table 1. We found 11 clusters of a larger size (≥5), eight formed by MSM and three by IVDU. It was noteworthy that one cluster was formed by 58 young subjects (median age of 33) with a median CD4+ T-cell count of 518 cells/μl. This cluster showed short branches in the ML tree (Fig 1), which indicated short times between infections.

**Table 1:** Demographic, clinical and virological data and phylogenetic estimates for the HIV-1 subtype B patients included in the most epidemiologically relevant clusters (≥ 5 patients).

HIV-1 clade	N	Risk factor	Sampling interval	Age (median, IQR)	Nationality	Viral Load (median, IQR) Log_10_	CD4+ T-cell count (median,IQR)	Bootstrap value (%)	Posterior probability	tMRCA (95% HPD)	μ (95% HPD)	Ancestral location	Ancestral location probability
I	11	IVDU	2005–2011	47 (46–48)	Spanish	4.23 (3.71–4.72)	330 (132.5–541)	76	0.94	1979(1976–1991)	3.4 x10^-3^ (1.5 x10^-3^–6.2 x10^-3^)	Motril	0.64
II	8	IVDU	2005–2011	40 (37–48.5)	Spanish	4.73 (3.85–5.36)	323.5 (142–442.8)	70	0.97	1984(1979–1994)	3.4 x10^-3^ (1.5 x10^-3^–6.1x10^-3^)	Motril	0.94
III	7	MSM	2005–2009	49 (44–54)	Spanish	4.84 (4.44–5.22)	373.5 (256.8–628.75)	96	0.99	1991(1983–1997)	3.6x10^-3^ (1.7x10^-3^–6.1x10^-3^)	Granada	0.99
IV	8	MSM	2009–2012	39.5 (32–48)	Spanish-Romanian	5.34 (3.8–5.5)	553 (491–849)	94	0.97	1995(1982–1995)	3.0x10^-3^ (1.3 x10^-3^–5.2x10^-3^)	Granada	0.96
V	5	IVDU	2005–2011	45 (37–53.5)	Spanish-Algerian	4.81 (2.98–5.59)	295 (185.5–551.5)	88	0.98	1989(1980–1996)	3.2 x10^-3^ (1.4 x10^-3^–5.2 x10^-3^)	El Ejido	0.84
VI	58	MSM	2007–2012	32.5 (28–39.3)	Spanish	4.42 (4.03–5.27)	518 (301–896)	95	0.99	1994(1987–1999)	2.9 x10^-3^ (1.3 x10^-3^–4.7 x10^-3^)	Granada	0.93
VII	7	MSM	2007–2012	39 (32.3–52.5)	Spanish-Argentinian-Ecuadorian	5.15 (4.41–6.21)	326.5 (241.5–847.25)	86	0.98	1985(1983–1998)	2.1 x10^-3^ (1 x10^-3^–3.4 x10^-3^)	Granada	0.99
VIII	7	MSM	2009–2011	38 (29.5–42.3)	Spanish	4.46 (4.28–5.04)	279.5 (8.25–598)	96	0.95	1993(1992–2002)	3.7x10^-3^ (2.0 x10^-3^–5.7 x10^-3^)	Granada	0.99
IX	5	MSM	2006–2012	43 (38–45.5)	Spanish	5.71 (4.67–6.32)	213 (86–542.5)	100	0.99	1997(1995–2005)	3.2 x10^-3^ (1.6 x10^-3^–5.2x10^-3^)	Granada	0.99
X	12	MSM	2007–2012	37.5 (30–44.3)	Spanish	5.17 (4.81–5.98)	331 (252.3–388)	99	0.99	2001(1998–2005)	3.5 x10^-3^ (1.5 x10^-3^–6.1x10^-3^)	Granada	0.99
XI	5	MSM	2005–2010	43 (34–45)	Spanish	4.36 (3.94–5.14)	325 (174.5–380)	99	0.99	1997(1994–2003)	2.3 x10^-3^ (1.3 x10^-3^–4.2 x10^-3^)	Granada	1

Table 2 shows the demographic characteristics of the patients, who were included, or not, in the transmission clusters. Statistically significant differences were observed between the patients included, or not, in the clusters. The association was more frequent in Spanish (p = 0.02) men (p< 0.001), MSM (p<0.001), younger than 35 years (p = 0.001) and with a CD4+ T-cell count above 350 cells/ul (p<0.001).

**Table 2:** Clinical, demographic and virological characteristics of the patients within or outside of transmission clusters obtained by phylogenetic analyses.

**Patients, n (%)**						
	Total	Inside Cluster	Outside Cluster	P value^a^	OR (95% CI)	P value^b^
	493(100)	234(100)	259(100)			
**Gender**				**<0.001**		
Male	404 (81.9)	209(89.3)	195(75.3)		Ref	
Female	89 (18.1)	25(10.7)	64(24.7)		0.312(0.184–0.531)	**<0.001**
Nationality (453)*				**0.020**		
Spanish	398 (87.9)	196 (89.9)	202 (86)		Ref	
Foreign	55 (12.1)	22 (10.1)	33 (14)		0.491(0.267–0.904)	**0.022**
Age (460)*				**0.001**		
<35	123(26.7)	78(35.3)	45(18.8)		Ref	
35–45	167(36.3)	72(32.6)	95(39.7)		0.480(0.299–0.771)	**0.002**
>45	170(37)	71(32.1)	99(41.4)		0.445(0.278–0.774)	**0.001**
**City**				0.082		
Almería	60(12.2)	26(11.1)	34(13.1)			
El Ejido	66(13.4)	31(13.2)	35(13.5)			
Granada	233(47.3)	112(47.9)	121(46.7)			
Motril	46(9.3)	30(12.8)	16(6.2)			
Jaén	88(17.8)	35(15)	53(20.5)			
**Sampling Year**				0.388		
2005	40(8.1)	16(6.8)	24(9.3)			
2006	41(8.3)	14(6)	27(10.4)			
2007	62(12.6)	29(12.4)	33(12.7)			
2008	48(9.7)	26(11.1)	22(8.5)			
2009	109(22.1)	58(24.8)	51(19.7)			
2010	45(9.1)	26(11.1)	19(7.3)			
2011	76(15.4)	30(12.8)	46(17.8)			
2012	72(14.6)	35(15)	37(14.3)			
Viral Load (456)*				0.070		
<10.000	94(20.6)	38(17.4)	56(23.5)			
10.000–100.000	205(45)	101(46.3)	104(43.7)			
>100.000	157(34.4)	79(36.2)	78(32.8)			
CD4 + T-cell count (427)*				**<0.001**		
<200	121(28.3)	38(19.2)	83(36.2)		Ref	
201–350	95(22.2)	43(21.7)	52(22.7)		2.079(1.185–3.646)	**0.011**
>350	211(49.4)	117(59)	94(41)		2.980(1.847–4.809)	**<0.001**
Risk factor (433)*				**<0.001**		
MSM	210(48.5)	156(66.7)	54(27.1)		Ref	
IVDU	91(21)	39(16.7)	52(26.1)		0.286(0.170–0.480)	**<0.001**
HTX	132(30.5)	39(16.7)	93(46.7)		0.271(0.170–0.432)	**<0.001**

^a^*P* value for the chi-square test.

^b^*P* value for the univariate logistic regression.

*Data available for the number of patients indicated.

In order to test the integrity of clusters, we retrieved from GenBank the five most closely related (at least 95% of genetic similarity) HIV-1 sequences to all the 234 study sequences included in the clusters using ViroBLAST (https://indra.mullins.microbiol.washington.edu/viroblast/viroblast.php). In a phylogenetic tree constructed with RAxML, we confirmed that none of the initially found clusters were broken down by adding these similar sequences from public databases, which ensures their status of real clusters circulating in east Andalusia (S1 Fig). It was noteworthy that sporadic GenBank sequences (particularly from other Spanish regions, but with limited available demographic information) formed part of some of east Andalusian lineages, which suggests linkage to other epidemics.

**Fig 2:**
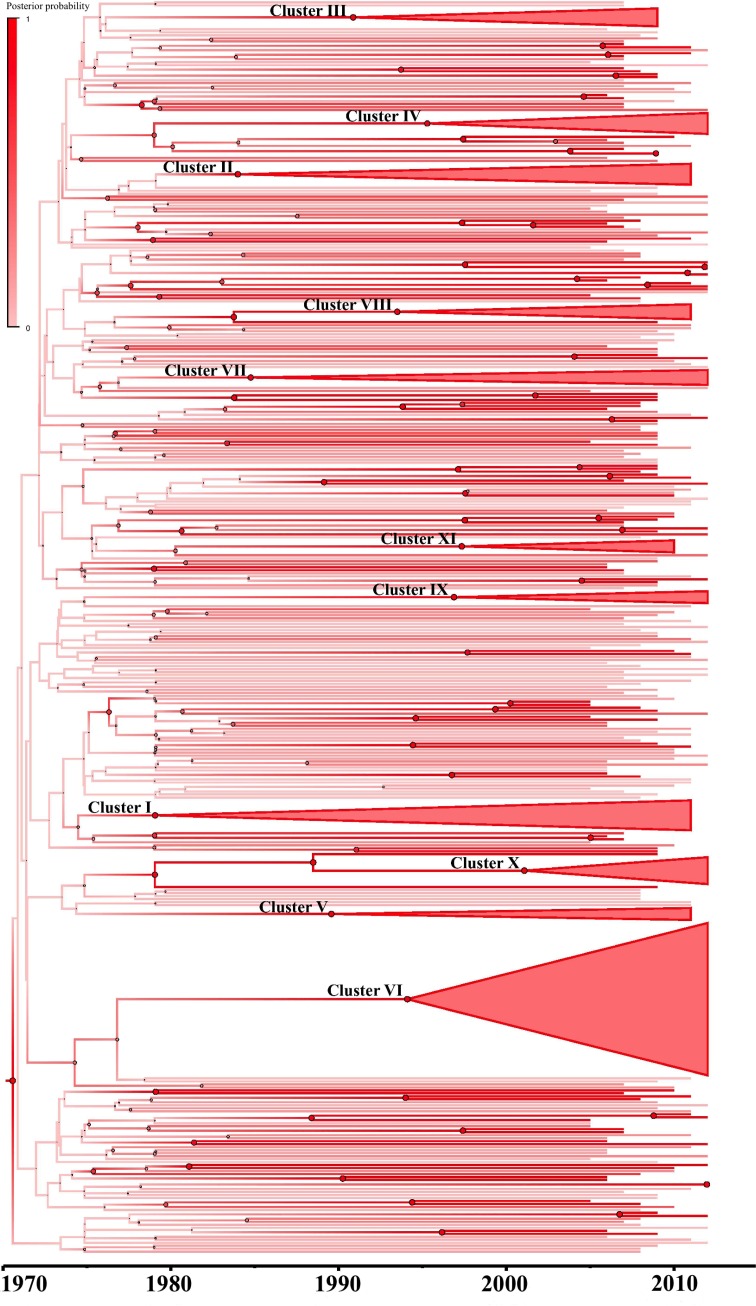
Maximum clade credibility tree generated by the Bayesian inference method using the BSP non-parametric model, as implemented in BEAST v1.7.5. Branches were drawn using a progressive posterior probability value of 0–1. The clusters previously defined through ML and the statistically highly supported nodes (pp >0.9) were defined on a time scale.

**Fig 3:**
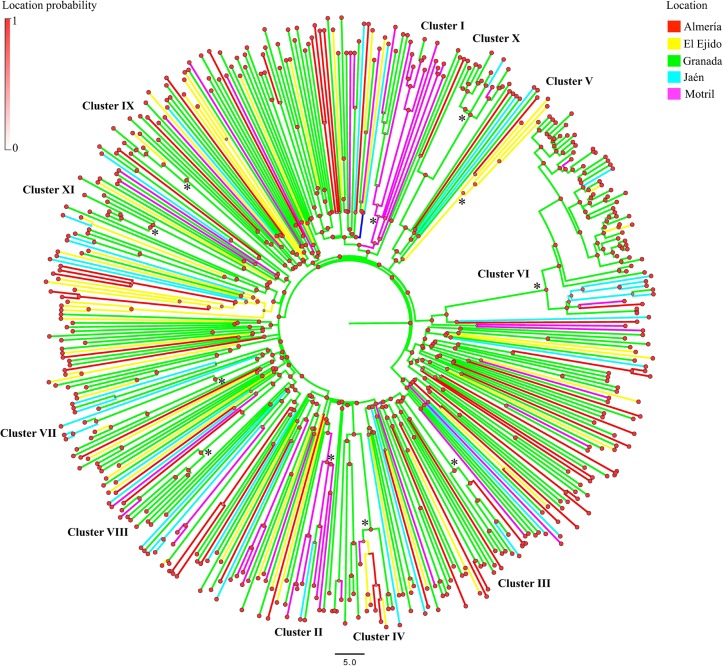
Bayesian maximum clade credibility phylogeographic tree of the HIV-1 Subtype B by the Bayesian discrete approach in BEAST v1.7.5. The upper left bar represents the gradient of location probability. Nodes were drawn using a progressive ancestral probability location value of 0–1. Colours of terminal and internal branches indicate the sampling location for each sequence and the most probable ancestral location for each clade, respectively. The clusters of greatest epidemiological interest (≥5 patients grouped) are indicated by an asterisk (*).

By analysing the PR+RT genomic region, with an uncorrelated lognormal clock and using the Bayesian Skyline Plot model, we obtained a mean evolutionary rate of 2.4 (95% credible region: 1.7–3.1) x 10^−3^ substitutions/site/year. The MRCA for subtype B in east Andalusia was estimated in 1970 (95%CI: 1965–1987). The Bayesian Maximum clade credibility (MCC) tree (Fig 2) shows the clusters in a temporal context. The inferred tMRCA and ancestral location posterior probabilities (>0.9 in all cases) for clusters are shown in Table 1. The tree represented in Fig 3 indicates the subtype B phylogeography in east Andalusia, while the most probable ancestor location and location probability of the most relevant clusters are provided in Table 1.

The oldest lineages found in east Andalusia originated in the late 1970s and the early 1980s in the coastal area of Motril, and were formed by intravenous drug users: Cluster I (location probability = 0.64) in 1979 (95%CI: 1976–1991) and Cluster II (location probability = 0.94) in 1984 (95%CI: 1979–1994). As shown in Table 1, most of the more epidemiologically relevant clusters were formed by MSMs, most of which originated in the 1990s, all in Granada, with location probabilities > 0.9.

**Fig 4:**
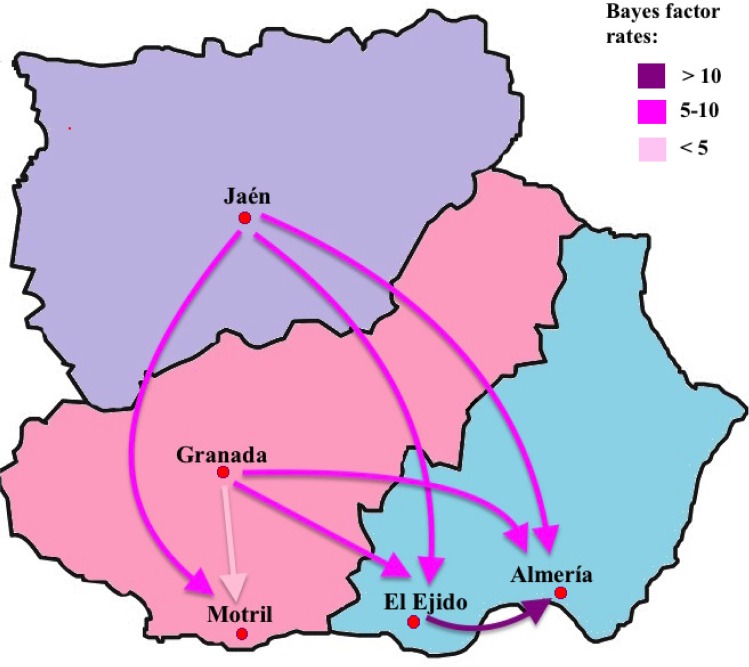
The most significant epidemiological links of HIV-1 subtype B dispersal in Andalusia. Only the epidemiological links supported by Bayes factor rates above 3 are indicated. The legend for the strength of the Bayes factor rates is shown on the upper right.

The Bayesian phylogeographic analyses of HIV-1 subtype B indicated that the ancestral lineages originated in Granada. From this city, the HIV-1 subtype B epidemic in Andalusia sequentially propagated to different peripheral areas of east Andalusia, such as the coasts of El Ejido, Motril and Almería, and eventually to the landlocked area of Jaén. The most significant (Bayes Factor>3) migration routes of HIV-1 subtype B showed directionality from the inland Granada and Jaén regions to the coastal areas of El Ejido and Almería (Fig 4).

**Fig 5:**
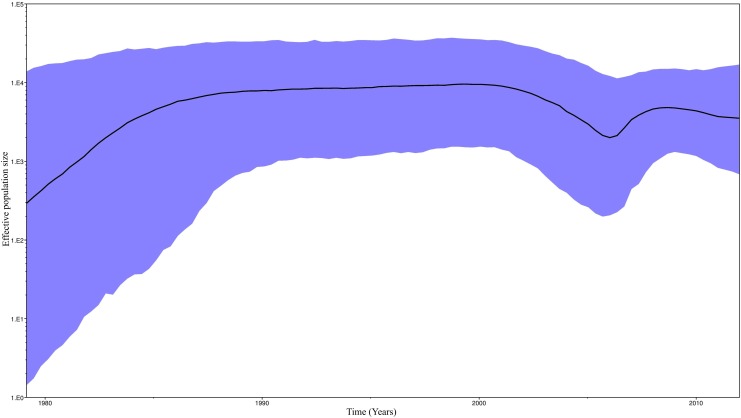
Demographic history of HIV-1 Subtype B in Andalusia. A demographic analysis was performed using the Bayesian Skyline Plot. The vertical axes represent the estimated effective population size on a logarithmic scale. The black line represents the median estimated for the effective population size with time. The blue area depicts the 95% HPD (high posterior density) confidence interval for this estimate.

**Fig 6:**
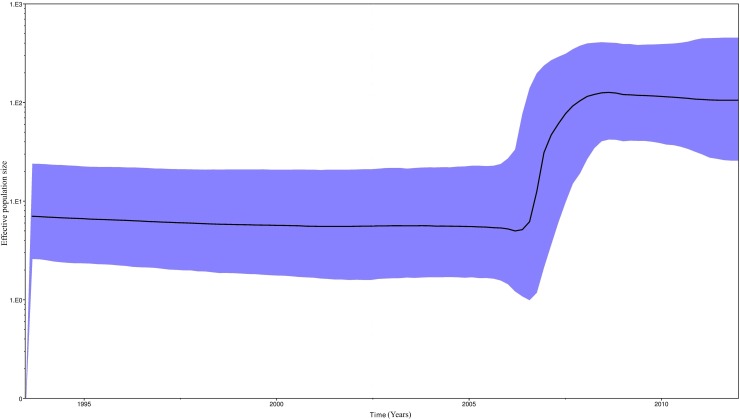
Demographic history of the major HIV-1 clade. A demographic analysis was performed using the Bayesian Skyline Plot. The vertical axes represent the estimated effective population size on a logarithmic scale. The black line represents the median estimated for the effective population size with time. The blue area depicts the 95% HPD (high posterior density) confidence interval for this estimate.

A Bayesian Skyline plot (Fig 5) was used to illustrate the dynamic growth of HIV-1 subtype B, and changes in the effective size of the epidemic in the last three decades were estimated. A demographic analysis of our population showed initial growth in the 1980s, which stabilised in the 1990s. In the first decade of this century, epidemic size became smaller, but once again increased during the 2005–2008 period to finally stabilise. Finally, we analysed the phylodynamic profiles of the major HIV-1 clade (Cluster VI) in east Andalusia (Fig 6). The exponential growth from 2005 may partially explain the increase in the effective population size of the epidemic HIV-1 subtype B during this period.

## 4. Discussion

More than half the HIV-1 subtype B-infected newly diagnosed patients in east Andalusia cluster in transmission cluster formed by five or more individuals. Most patients are young MSM with a high CD4+ T-cell count. These data suggest that a considerable number of patients present acute and recent HIV-1 infection.

The estimated mean evolutionary rate of subtype B epidemics (2.4 (95%CI: 1.7–3.1) x 10^−3^ substitutions/site/year) was similar to that reported in other studies (1-3x10^-3^ substitutions/site/year) [38–40]. This rate tended to be higher for the lineages formed largely by MSMs and IVDUs, which suggests greater transmission efficiency and faster subtype B evolution in the groups at risk of HIV-1 infection [41].

Our study, like other European surveys, [39, 42–44] confirmed that the subtype B epidemic in east Andalusia started by diversifying before the first AIDS diagnosis was made in Spain in 1981 [45]. As in other studies of this kind, the dates estimated with phylodynamics approaches that were sensitive to old lineages disappeared before our sampling times or past large waves of new HIV-1 introductions. In addition, this diversification might have started elsewhere.

We demonstrated how the effective population size of subtype B HIV-1 in east Andalusia underwent exponential growth from the early 1980s to the early 1990s, which is in line with reports from other countries [43, 46–49]. Our study suggests that the early East Andalusian lineages originated in the 1980s among intravenous drug users in coastal areas. Epidemic growth stabilised in the early 1990s (Fig 6), when prevention measures were first implemented in Spain [50]. Since then, the transmission pattern in Spain has gradually evolved from IVDUs to MSMs [45]. This partially explains why we detected an emergence of clustering among the MSMs for these dates, which fell in line with increased high-risk behaviour by MSMs reported in Europe for these years [51–53]. From this period, access to highly active antiretroviral treatment (HAART) in 1996 in Spain has helped reduce the effective population size for HIV-1 among infected patients [54–57]. However from 2005, the subtype B epidemic in east Andalusia underwent new exponential growth, which indicates that dedicated preventive measures to avoid growth must be implemented and maintained. Multiple studies [19] have interpreted the timing of changes in phylodynamic patterns in the context of other changes. Hence we believe that this type of studies may serve as a tool to implement preventive strategies.

An especially important lineage in our population was cluster VI, which was formed by 58 individuals with MRCA, and appeared in 1994 (95%CI: 1987–1999). New subjects continued to enter this cluster until the end of our study period. Indeed young MSM, with high CD4+ T-cell counts at the time of diagnosis, made up this cluster, which suggests recent infection.

Our phylogeographic study showed how HIV-1 subtype B spread mainly from inland regions to coastal cities, and from El Ejido to the city of Almería in east Andalusia. This was perhaps motivated primarily by a higher proportion of patients infected with HIV-1 to these areas while visiting coastal areas and, secondly, also by many migrants (25/55, 45%) who concentrate in El Ejido, where approximately a third of patients are migrants (25/66, 38%; S2 Fig) who come mainly from North Africa (15/25, 60%) (data not shown). For a number of years, this area has been a gateway to African migration into Spain and Europe, and has contributed to numerous transmission events.

Despite the fact that this work included all the available HIV-1 subtype B sequences generated for resistance testing during the study period and in the study area, the likely existence of undetected and undiagnosed HIV-infected subjects prevented us from detecting all the viral lineages that circulate in east Andalusia. In addition, this study did not take into account migration from beyond this region because we chose to focus on the net trends throughout the region, regardless of sporadic imports or exports. It would be most unlikely that all the subtype B lineages of foreign origin were circulating in our region without been noticed or sampled. We also intended to uncover the long-term well-established lineages that circulate in the region for the first time. Future studies will be designed to uncover the relationship of the east Andalusian HIV-1 epidemic to other regions or countries. Another aspect to consider is that the phylogenetic cluster definition vastly varies in the literature. Most studies have used node statistical support, genetic distance or time depth [58]. We relied on a bootstrap value in the ML analysis, combined with posterior probabilities in the Bayesian approach. This combination is used to maximise the probability of finding regional epidemiologically relevant phylogenetic lineages.

Several European cohorts have described the recent emergence of HIV-1 clusters, mainly among young MSMs [59–61]. In Spain, Yebra et al [62] reported a higher inclusion of MSMs into transmission clusters, and Cuevas et al [63] described a higher proportion of MSM clustering in the Basque country, with an important cluster that included 12 patients with the T215D revertant. Interestingly, an expansion of this cluster has been recently reported by Vega et al [64], and Patiño-Galindo et al [65], who have added the origin and diversification of clusters in the 1990s. These findings may contribute to explain the growth in new infections in recent years [66,67], and stress the need to increase access to HIV testing to lower transmission rates in high-risk populations.

In summary this is the first study of the evolutionary history of HIV-1 subtype B infection, phylodynamics and phylogeography in east Andalusia. We detected long-term lineages among high-risk populations. We present data that are extremely important to help develop preventive strategies in our scenario. We also showed how phylodynamics can be used to evaluate model occurrence of new HIV-1 outbreaks. We mapped subtype B HIV-1 migration in east Andalusia. Both phylogeographic and phylodynamic contributions should be considered when healthcare managers develop appropriate prevention strategies.

## Supporting Information

S1 Fig Maximum likelihood tree of the sequences included in the detected east Andalusian lineages (n = 234), together with the most closely related HIV-1 sequences from GenBank. Tip labels are depicted in blue for the east Andalusian sequences (and indicate the sampling date and region) and in black for the GenBank sequences (and indicate subtype, country, accession number and sampling date). Lineages are highlighted.
(EPS)

S2 Fig Prevalence of HIV-1 infected patients in Eastern Andalusian cities (Spanish vs foreign). Prevalence based on all patients with nationality data available (n = 453).
(EPS)
